# Hypokalemia Leading to Postoperative Critical Arrhythmias: Case Reports and Literature Review

**DOI:** 10.7759/cureus.8149

**Published:** 2020-05-16

**Authors:** Syed Muhammad Ali, Nisar Shaikh, Fakhar Shahid, Amjad Shah, Hafiz Bilal Zafar

**Affiliations:** 1 Acute Care Surgery, Hamad General Hospital, Doha, QAT; 2 Surgery, Weill-Cornell Medical School, Doha, QAT; 3 Surgical Intensive Care, Hamad Medical Corporation, Doha, QAT; 4 General Surgery, Hamad Medical Corporation, Doha, QAT; 5 Surgery, Hamad Medical Corporation, Doha, QAT

**Keywords:** perioperative arrhythmias, hypokalemia, cardiopulmonary resuscitation, potassium chloride

## Abstract

Perioperative arrhythmias can develop due to many reasons, rarely life-threatening, but hypokalemia plays an important role in their development. We report two cases of severe postoperative hypokalemia leading to ventricular fibrillation (VF).

Case 1: A young healthy lady developed perioperative severe hypokalemia leading to repeated episodes of VF requiring cardiopulmonary resuscitation (CPR), direct current (DC) shock and anti-arrhythmic therapy, apart from rapid replacement of intravenous potassium. She recovered fully without any neurological or cardiac sequelae.

Case 2: A 78-year-old male patient, a known case of hypertension controlled with medications developed postoperative repeated VF due to hypokalemia requiring 210 mmol of potassium chloride, antiarrhythmic therapy, DC shock, and CPR. He recovered, but complicated into acute myocardial infarction requiring therapy.

Perioperative severe hypokalemia can lead to life-threatening cardiac arrhythmias. Early recognition and aggressive correction are essential for better outcomes.

## Introduction

Sudden or acute onset life-threatening perioperative arrhythmias are a rare clinical entity in noncardiac surgical patients but are common phenomena in cardiothoracic surgery patients [1]. Electrolyte imbalance, particularly hypokalemia, is a possible underlying cause for these arrhythmias. Hypokalemia is classified as moderate when serum potassium levels are 2.5-3 mmol/L (reference range, 3.5-5 mmol/L) or severe when serum potassium level is lower than 2.5 mmol/L. We report two cases of severe hypokalemia leading to life-threatening cardiac arrhythmias in the postoperative period. An overview of these cases was initially presented via an abstract at the Qatar Critical Care Conference Proceedings [2]. The full details of the cases are presented herein.

## Case presentation

Patient Case 1

**Figure 1:**
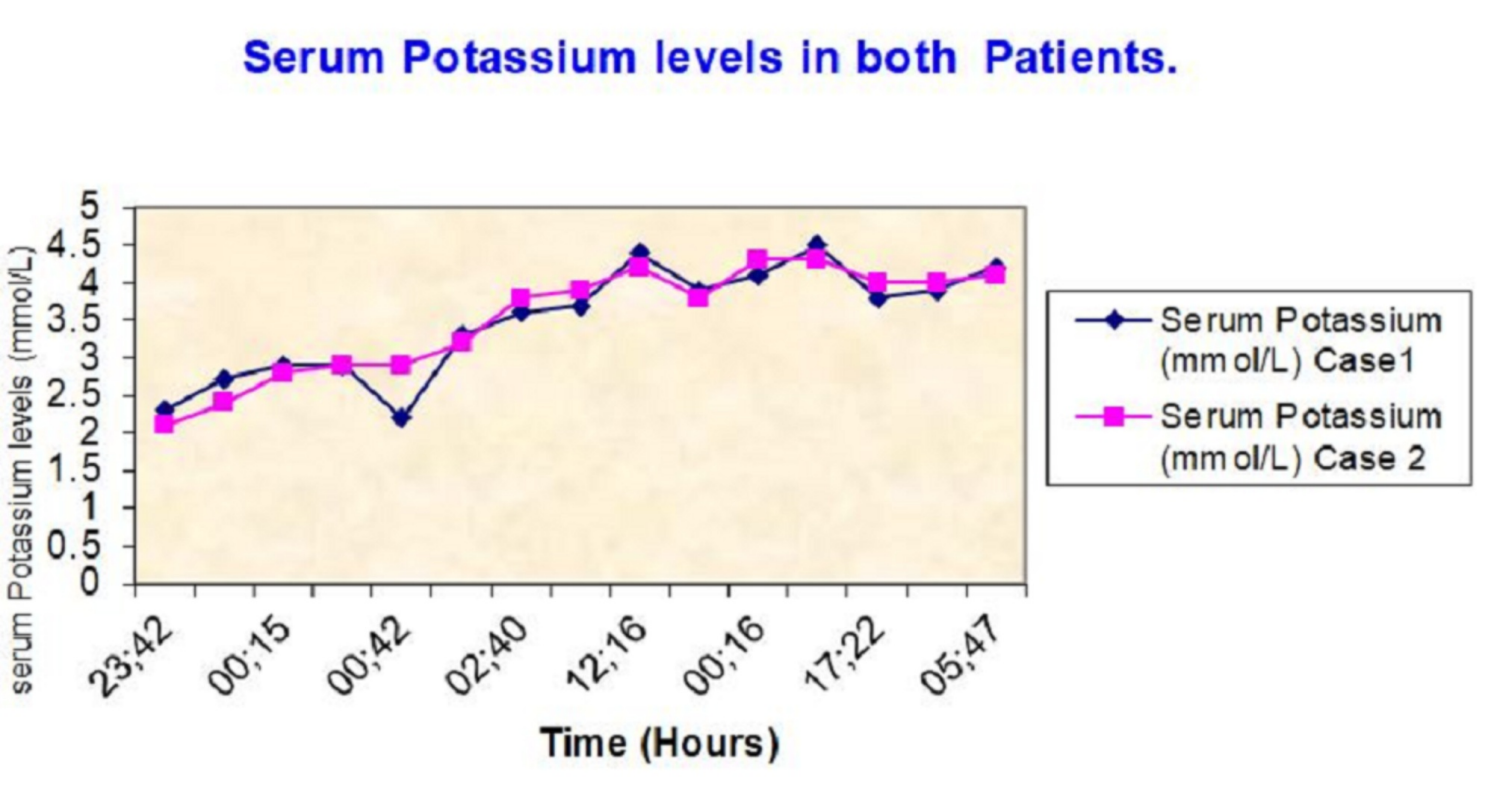
Serum potassium levels of both patients over time.

A 30-year-old healthy woman had emergency laparoscopic cholecystectomy and appendectomy. She reported a history of bronchial asthma untreated for the past three years. The preoperative and intraoperative periods were uneventful. Her preoperative potassium level was 3.7 mmol/L. After 18 hours of surgery, she suddenly developed palpitation followed immediately by cardiac arrest. She entered ventricular fibrillation (VF) and received cardiopulmonary resuscitation (CPR) and direct current (DC) shock that led to sinus rhythm. She was shifted to the ICU, intubated, and started on assisted ventilation. In the ICU, her serum electrolytes showed severe hypokalemia (serum potassium, 2.2 mmol/L; Figure 1). She was immediately started on 20 mmol of potassium chloride (KCl) over 30 minutes through a central venous catheter under monitoring, and KCl was added to the intravenous fluids. In the next 36 minutes, she had four episodes of VF requiring DC shock and CPR. She received an amiodarone infusion along with continuous KCl supplementation and calcium gluconate (2 g). She received 100 mmol of KCl in six hours and a total of 220 mmol of KCl in 24 hours, after which she became stable and showed signs of cardiovascular stability. She was extubated after 48 hours when her echocardiogram showed no pathological changes, and no abnormalities were detected on cardiac conduction studies (i.e., electrophysiological studies). She recovered smoothly with no neurological deficit. She was discharged home on day 12 and monitored via follow-up at the outpatient clinic where she was found in good health.

Patient Case 2

A 78-year-old man with a history of hypertension controlled with angiotensin-converting enzyme inhibitors, with normal preoperative cardiac workup including ECG, was moved to the ICU after laparoscopic cholecystectomy for observation. The patient remained intubated after the surgery, and his preoperative serum electrolytes were within the reference range (serum potassium, 3.8 mmol/L). In the ICU, after one hour, he started to develop tachycardia, then went into pulseless ventricular tachycardia (VT), and needed defibrillation. His serum electrolytes indicated severe hypokalemia (2.4 mmol/L; Figure 1). He was started on rapid correction with KCl through his central venous catheter and supplementation of KCl in intravenous fluids. After 10 minutes, he developed VF requiring DC shock and a bolus of amiodarone. Over the next 20 minutes, he had three more episodes of VF requiring CPR and DC shock.

In the next six hours, he received 90 mmol of KCl to attain serum potassium of 3.7 mmol/L. A total of 210 mmol of KCl was given in 24 hours. His blood glucose was within the reference range during the episodes of VF. He was extubated after 24 hours. His echocardiogram showed anterior wall motion abnormality with an ejection fraction of 52%. He was started on aspirin, clopidogrel, and atorvastatin. He was transferred to the ward on day three and discharged home after one week. He was monitored via follow-up in the outpatient clinic and showed no abnormality.

## Discussion

Potassium is essential for the maintenance of cellular polarization as well as the transmission of electrical impulses through the myocardium. Hence, drastic changes in serum potassium levels (i.e., hypokalemia or hyperkalemia) will lead to arrhythmias [3]. Although cardiac arrhythmias are common in the perioperative periods, they are rarely life-threatening and usually do not require CPR and antiarrhythmic therapy. Perioperative VF is common in cardiac patients with hypokalemia [4].

Neither of our patients had any known coronary arterial disease, but they still developed life-threatening VF due to severe hypokalemia. There are various etiological causes for the development of severe perioperative arrhythmias such as gastrointestinal or renal loss of potassium, hyperglycemia, and medications like salbutamol. According to Hollifield and Slaton, in patients receiving high-dose thiazides, the incidence of cardiac arrest was three to six times greater than in nonthiazide receiving patients, and this risk significantly reduced if potassium-sparing diuretics were added [5]. Hollifield and Slaton found no association with cardiac arrhythmias in patients with serum potassium levels ranging from 3.5 to 5 mmol/L. However, when levels fell below 3 mmol/L or elevated above 5.2 mmol/L, an invariant association with cardiac arrhythmias requiring CPR was found [5].

Walsh et al. analyzed prospective cohort studies of 6253 noncardiac patients [6]. Postoperative cardiac arrhythmias occurred in 7.84% patients, atrial fibrillation was the most common arrhythmia (4.4%), and VF was very rare, accounting for only 0.02% of cases evaluated [6]. Walsh et al. found that older patients, male gender, asthma, history of arrhythmias, and valvular heart diseases were the preoperative risk factors that increased the incidence of postoperative arrhythmias [6]. Another study by Walsh et al. reported that postoperative arrhythmias were common after cardiothoracic and colorectal surgery; one-third of these patients had abnormal magnesium, sodium, and potassium levels [7]. In both of our patients, however, no causative factor could be found.

Perioperative arrhythmias increase the incidence of myocardial infarction with a relative increased risk of 4.2% [8]. In 4.3 per 10000 patients, these arrhythmias lead to perioperative cardiac arrest [9]. The management of perioperative arrhythmias starts from recognizing the problem and diagnosing arrhythmias and their hemodynamic effects. Recognizing the type of arrhythmias is critical for an appropriate therapeutic approach as the treatment will be either electrical, pharmacological, or a combination of both. Physicians should look for the precipitating factors or underlying etiological reasons for arrhythmia, particularly electrolyte disorders (e.g., hypokalemia, hypomagnesemia, or hyperkalemia) and the presence of sepsis or cardiovascular disorder. In particular, hypokalemia should be detected early, and such patients started with intravenous replacement with KCl given that serum levels <3 mmol/L are associated with significantly increased risk of ventricular fibrillation [4].

If a patient suddenly enters cardiac arrest, resuscitation should be initiated as per advanced life support (ALS) guidelines. Cardiac rhythm (in this situation) is divided into shockable rhythms like VT and VF or nonshockable states like asystole and pulseless electrical activity. It is of vital importance in cases of shockable rhythm because early defibrillation will improve the patient’s outcome [10]. If severe hypokalemia is encountered, causing cardiac arrest, correction of acidosis with sodium bicarbonate administration must be done with caution as it will further decrease the serum potassium level and may lead to refractory VF.

Pharmacological agents used in VF is amiodarone, which should be given preferably through a central venous line as it has a low incidence of Torsade’s de Pointes, which helps maintain sinus rhythm. Once sinus rhythm is achieved with DC shock on patients with VT or VF, amiodarone improves survival [11].

## Conclusions

Hypokalemia is a risk factor for cardiac arrhythmias in the perioperative period. VF and cardiac arrest rarely occur in the perioperative period due to severe hypokalemia. For patients with perioperative VF, early DC shock and amiodarone improve survival. If a patient has perioperative VF/VT and requires CPR due to severe hypokalemia, physicians should use sodium bicarbonate with caution, given that it further lowers serum potassium levels. Perioperative cardiac arrhythmias increase the risk of myocardial infarction and perioperative cardiac arrest.
